# Healthcare professionals’ perspectives on usefulness, acceptability and implementation conditions of socially assistive robots in France: a cross-sectional survey and cluster analysis

**DOI:** 10.3389/fdgth.2026.1802396

**Published:** 2026-06-04

**Authors:** Lauriane Blavette, Sébastien Dacunha, Anastasiia Bondarenko, Matthieu Piccoli, Anne-Sophie Rigaud, Maribel Pino

**Affiliations:** 1 Service Gériatrie 1&2, Centre Mémoire de Ressources et Recherches Île-de-France-Broca, Assistance Publique—Hôpitaux de Paris, Hôpital Broca, Paris, France; 2 Unité Mixte de Recherche-Santé 1144, Optimisation Thérapeutique en Pharmacologie (OTEN), Université Paris Centre, Paris, France; 3 Broca Living Lab, Hôpital Broca, Paris, France; 4 Université Paris Cité, Faculté Sociétés et Humanités, Paris, France

**Keywords:** ethical issues, healthcare professionals, implementation conditions, perceptions, socially assistive robots, survey, technology acceptance, usefulness

## Abstract

**Introduction:**

In healthcare, socially assistive robots are increasingly used for logistical, assistive, and psychosocial purposes, raising ethical, social, and organizational questions. In these contexts, professionals’ acceptability varies by use case, perceived risk, and care setting. Understanding how healthcare professionals evaluate these technologies is essential for anticipating their large-scale integration into health systems and its implications for workforce organization and equity of access.

**Methods:**

This cross-sectional survey in France examined healthcare professionals’ perceptions of socially assistive robots, focusing on perceived usefulness, acceptability, and implementation-related factors. A self-administered 48-item questionnaire covered sociodemographic characteristics, knowledge of robots, perceived usefulness across use cases, importance of implementation factors, and acceptability and intention to use. Data were analyzed using descriptive statistics, non-parametric tests, and principal component analysis with hierarchical clustering to identify attitudinal profiles.

**Results:**

A total of 148 healthcare professionals participated, 77% reporting prior knowledge of robots. Perceived usefulness was generally high, particularly for physical tasks and recreational support, while therapeutic mediation and feeding were rated lower. Ethical, organizational, and regulatory factors were rated as very important, and acceptability was higher for general use than for personal clinical practice. Cluster analysis identified three attitudinal profiles characterized by low, moderate, and high acceptability.

**Discussion:**

Healthcare professionals expressed generally favorable but selective attitudes toward socially assistive robots, mainly valuing logistical and organizational support and remaining more cautious about therapeutic and psychosocial uses. Acceptability appeared conditional and context-dependent, linked to perceived usefulness, safeguards, and prior knowledge rather than professional or sociodemographic characteristics. These findings highlight the need for public-health and implementation strategies combining clear ethical and legal frameworks, training, and context-specific integration, and the relevance of longitudinal mixed-method studies to examine how attitudes and adoption evolve with real-world use.

## 1 Introduction

Healthcare systems are undergoing sustained structural change, driven by population aging, the rising prevalence of chronic conditions, and increasing fragmentation across care pathways (1, 2). These transformations coincide with persistent workforce shortages, high staff turnover, and growing professional burnout, further intensified during periods of systemic disruption such as the COVID-19 pandemic and recurrent seasonal influenza (3–5). Taken together, these developments reflect broader organizational and socio-economical constraints that influence how care is delivered, coordinated, and sustained over time (6).

Within this context, digital health technologies, artificial intelligence (AI), and socially assistive robots (SARs) are increasingly introduced as part of organizational strategies intended to support healthcare professionals and maintain care quality. In practice, these technologies are often deployed in relation to operational objectives such as improving efficiency and quality of services, reducing physical workload, addressing resource constraints, or reorganizing care processes (7–10).

The integration of new technologies in healthcare has often been examined through technology acceptance frameworks that aim to explain how individuals evaluate and adopt innovations in professional contexts. Among the most widely used models, the Technology Acceptance Model (TAM) and its extensions propose that technology adoption is largely influenced by perceived usefulness and perceived ease of use, which shape users’ attitudes and intention to use a system (11, 12). In healthcare settings, these models have been complemented by broader implementation frameworks that emphasize organizational, ethical, and contextual factors influencing the integration of new technologies into routine practice (13, 14). Overall, these approaches show that the adoption of a technology depends primarily on how users assess its relevance, acceptability and feasibility in real-world healthcare settings.

In this perspective, several interrelated constructs are commonly examined when studying the adoption of healthcare technologies. Perceived usefulness refers to the degree to which individuals believe that a technology can enhance their work performance or, in the healthcare context, support clinical objectives (11). Acceptability refers more broadly to users' cognitive and emotional responses to a product, or intervention, and the extent to which they consider it appropriate or are willing to integrate it into practice (15). Finally, implementation conditions refer to the organizational, ethical, regulatory, and practical factors that influence whether a technology can be effectively deployed and sustained in real-world healthcare settings. These factors may include training, workflow integration, regulatory frameworks, cost, and ethical safeguards.

SARs constitute a specific category of service robots designed to assist users primarily through social interaction rather than through physical manipulation. These robots aim to support communication, motivation, emotional engagement, or daily activities through speech, gestures, or other social cues (16). In healthcare contexts, SARs are implemented in a range of clinical and care environments, including hospitals, rehabilitation units, long-term care facilities, and geriatric services. Their applications encompass psychosocial support, cognitive stimulation, communication facilitation, and in some cases support for daily activities and care organization (17, 18). However, uptake of SAR-based interventions varies considerably across settings, highlighting the role of institutional contexts, regulatory frameworks, and professional practices in determining how SARs are introduced and integrated into routine care.

The introduction of SARs into everyday healthcare work is often accompanied by ethical, social, and organizational issues that influence professionals' evaluations and use of these technologies. In the field of geriatric care, for instance, when SARs are used within psychosocial care practices, ethical concerns often emerge, including issues of dignity, autonomy, consent, privacy, and potential changes in therapeutic relationships (19–21). Qualitative studies examining the use of SARs with older adults with dementia illustrate a range of ethical issues associated with the use of SARs in healthcare. Among these, attachment to a SAR (i.e., the development of emotional reliance or preferential engagement with the robot) is generally not considered problematic in itself, but may raise ethical concerns in certain situations, particularly when it interferes with individuals' routines, emotional regulation, or well-being, leading care teams to exercise ethical judgment in reassessing the appropriateness of continued use and, in some cases, to limit or discontinue the intervention (22). From a safety and accountability perspective, automated systems introduce new modes of failure and responsibility that challenge existing mechanisms for monitoring system behavior, managing risk, and responding to adverse events, particularly when AI is involved (10, 23). From an organizational perspective, research conducted in institutional dementia care settings indicates as well that implementing pet robots generates additional, often invisible work for healthcare staff, including charging, cleaning, hygiene management, storage, and ongoing monitoring of residents' responses (22).

Previous research further indicates that healthcare professionals' acceptability of the use of SARs varies according to the type of task for which the robot is deployed (e.g., logistical support, physical assistance, psychosocial interaction), the perceived risks associated with its use for patients (e.g., safety concerns, psychological risks, impact on autonomy), and its compatibility with existing workflows and care routines (e.g., time constraints, coordination within care teams, integration into daily practices) (24, 25, 57). Higher acceptance is typically reported for robotic applications aimed at reducing physical workload, preventing occupational injury, or supporting logistical tasks, whereas lower acceptance is observed for functions involving clinical decision-making or sustained relational interaction (26, 27). Acceptance is also influenced by prior exposure to technology, access to training, organizational support, and perceived alignment between technology functions and concrete clinical needs (28–30). Across studies, acceptance of SARs is consistently associated with alignment with professional values and ethical norms, control over system use, and clarity regarding professional responsibility; these factors are often reported by healthcare professionals as playing a greater role in adoption decisions than assessments of technical capabilities of SARs alone (21, 31, 32).

Despite a growing body of work in healthcare robotics, important gaps remain in the examination of ethical, social, and organizational dimensions related to the acceptance and use of SARs. Much of the literature has primarily emphasized individual-level perceptions of usefulness and ease of use, with comparatively less attention devoted to how SARs are globally evaluated by healthcare professionals within the organizational and ethical contexts of everyday practice (17, 26). While existing studies address these dimensions in different ways (29, 33, 56) few adopt an integrated perspective that simultaneously considers perceived use cases, implementation conditions, ethical considerations, and contextual acceptability across diverse professional roles.

Finally, as AI and robot-based technologies continue to evolve and become increasingly present in healthcare and everyday life, both technological capabilities and healthcare professionals’ attitudes toward their use are likely to change over time (34, 35). In line with this perspective, Barbul et al. (36) identify generational differences in perceptions of robots and AI, with younger generations generally displaying greater openness toward human-like robotic technologies. The authors relate these differences to variations in technological exposure and digital socialization, illustrating how attitudes toward AI and robotics may evolve over time as these technologies become more widespread in everyday life. Extending these observations, previous work has shown that healthcare professionals' views toward SARs, digital, and AI-based technologies in healthcare are influenced not only by professional experience but also by broader social exposure to digital technologies, individual and cultural factors, and increasing familiarity with technology-mediated care practices (37–39). Together, these findings suggest that professional attitudes toward SARs and related technologies are dynamic rather than fixed. In this context, treating acceptability and implementation conditions as stable risks overlooking how perceptions of potential use cases, perceived risks, and practical constraints may evolve as technologies become more visible, accessible, and progressively normalized.

Survey-based research offers a relevant means of capturing healthcare professionals' perspectives on SARs and other digital and AI-based technologies at a given point in time across key dimensions such as perceived usefulness, ethical considerations, acceptability, and implementation-related factors. It also enables the identification of attitudinal profiles by showing how perceptions of usefulness, levels of acceptability, and the importance attributed to implementation conditions tend to cluster among professionals. Similar survey approaches have been used in healthcare robotics to document heterogeneity in attitudes toward robotic technologies; for instance, a cross-sectional online survey conducted among the Chinese general population (*n* = 428) identified distinct attitudinal profiles toward robots in healthcare (optimistic, neutral, and ambivalent) and reported associations between greater familiarity with digital technologies and more favorable perceptions (40).

Survey methods have also been applied to healthcare professionals specifically. Carradore et al. (41) conducted a survey among Italian healthcare professionals (*n* = 302) to examine attitudes toward SARs, reporting variations according to sociodemographic characteristics, work-related factors, and interest in technological developments, despite limited direct experience with robotic systems. In addition, Papadopoulos et al. (38, 39) carried out a large international online survey involving healthcare and social care professionals (*n* = 1,284) from 18 countries, focusing on perceived training needs related to SARs and documenting professionals' views on knowledge gaps, preparedness, and implementation conditions across healthcare contexts.

Taken together, these studies underscore the relevance of survey-based approaches for capturing diversity in attitudes, implementation-related considerations, and professional perspectives on SARs at specific moments in time and within specific geographic and healthcare-system contexts. By examining multiple dimensions simultaneously and at scale, surveys complement qualitative and experimental research by providing a broad overview of how SARs are currently understood and evaluated in routine practice, and, more generally, cross-sectional surveys are widely used in healthcare technology adoption to identify determinants of healthcare professionals' openness to and adoption of innovations as perceived at a given point in time (30). This perspective is particularly relevant for public health, given ongoing debates on workforce sustainability, care quality, and equitable access to SAR-based interventions.

This study adopts an exploratory approach to examine healthcare professionals' perceptions of SARs and the factors they consider important for their potential implementation in healthcare settings. The objective of this study is therefore to examine healthcare professionals' perceptions of SARs across a range of real-world clinical contexts in France, using a cross-sectional survey design. Specifically, the study aims to: (1) examine perceived usefulness across different healthcare use cases involving SARs; (2) assess professionals' evaluations of key implementation-related factors, including clinical relevance, ethical, organizational, economic, and legal considerations; (3) explore overall and context-specific acceptability of, and intention to use, SARs in healthcare practices, as well as their associations with professional role, experience, and prior exposure to this technology; and (4) identify distinct profiles of healthcare professionals based on patterns of prior knowledge of SARs, perceived usefulness, acceptability, and evaluations of implementation-related factors.

## 2 Methods

### 2.1 Participants

#### 2.1.1 Overview

Data collection was conducted between September 2023 and September 2025 using a self-administered questionnaire available in both online and paper formats. This relatively extended period reflects the exploratory nature of the study and the need to reach healthcare professionals across diverse care settings and professional roles. Because SARs remain relatively uncommon in many healthcare environments, a longer recruitment period was necessary to obtain a sufficiently large and heterogeneous sample. Dissemination primarily targeted healthcare professionals working in settings where SARs are currently discussed or considered, including hospital, rehabilitation unit, geriatric, pediatric, and medico-social contexts.

The online version of the questionnaire, hosted on the SurveyMonkey (42) platform, was disseminated through professional mailing lists and social networks (e.g., LinkedIn, Facebook). Posters containing a QR code linking to the survey were also displayed in healthcare settings. In parallel, on-site data collection was conducted during visits to hospitals in the Paris region and at healthcare-related events, where participants were invited to complete a paper version of the questionnaire, which was distributed and collected directly by the research team. Because the survey was circulated through open online channels, social networks, and posters with QR codes, the total number of professionals actually exposed to the invitation could not be estimated, and a response rate could therefore not be calculated.

Before completing the survey, all participants received an information sheet describing the study objectives, the voluntary nature of participation, confidentiality safeguards, and data protection procedures. Informed consent was obtained electronically for the online version and in writing for the paper format. Participation was anonymous, and no personally identifiable information was collected. To reduce the risk of duplicate participation, the online questionnaire was configured to allow only one submission per device (e.g., computer, tablet, smartphone), and participants were informed that the survey could be completed only once. During on-site data collection, paper questionnaires were distributed and collected directly by the research team, which limited the likelihood of duplicate responses.

#### 2.1.2 Ethical considerations

The study was approved by the French national ethics committee (Comité de Protection des Personnes, Ouest II, Maison de la Recherche Clinique—centre hospitalier universitaire Angers, institutional review board: 2021/20) and was fully compliant with the general data protection regulation (data protection officer reference: 20210114153645, AP-HP register).

#### 2.1.3 Sociodemographic characteristics

A total of 148 healthcare professionals participated in the study. Among them, 76.7% (*n* = 112) were women, 23.6% (*n* = 35) were men, and 0.7% (*n* = 1) selected another gender identity or preferred not to specify. The mean age of participants was 40 years (SD = 13.64; range: 19–79).

To facilitate interpretation, professions were grouped into ten functional categories reflecting roles within health and social care settings: (1) medical professions, including physicians, cardiologists, and pharmacists; (2) psychology, comprising psychologists and neuropsychologists; (3) nursing, including registered nurses and nurse coordinators; (4) care assistance, grouping hygiene technicians, childcare assistants, nursing assistants, and biomedical technicians; (5) rehabilitation and reeducation, including occupational and psychomotor therapists, physiotherapists, podiatrists, and adapted physical activity instructors; (6) management and leadership, covering healthcare managers, project managers, engineers, and administrative supervisors; (7) administrative and front-office staff, including medical secretaries, administrative staff, and reception officers; (8) dietitians; (9) students in health-related fields; and (10) social work professionals.

Students in health-related fields, were considered as a distinct category to differentiate individuals still in training and not yet fully integrated into a professional role, as their exposure to clinical environments, care practices, and healthcare technologies differs from that of practicing professionals.

Table 1 presents the distribution of participants across professional categories, including professional experience, knowledge of social and assistive robots, and previous exposure to robots in the work environment and in their own individual professional practice.

**Table 1:** Overview of the instrument domains and corresponding content.

Survey dimension	Content and description	Modality of answer
Section I: Sociodemographic and professional characteristics (7 items)	Information on participants’ sociodemographic and professional characteristics (gender, age, profession, years of experience, and workplace setting)	Open
Section II: Previous experience with SARs (4 items)	Prior exposure to SARs, including social robots, assistive robots, or both, was assessed. This section also examined participants’ prior knowledge of SAR use within their work environment, as well as any previous use of SARs in their own clinical practice. When applicable, participants were asked to specify the robot models encountered or used	Y/N question, and open
Section III: Perceived usefulness by use case (13 items) and preferred robot types (1 item)	Participants rated the perceived usefulness of SARs across a range of use cases, including activities of daily living, mobility, cognitive support, and communication. This section also assessed participants’ preferences regarding the types of robots they would be most inclined to use in healthcare settings	10-point Likert scale (1 = very low, 10 = very high) and multiple choise
Section IV: Implementation-related factors (21 items)	Participants evaluated the importance of factors related to the deployment of SARs in healthcare (e.g., cost, ease of use, ethical considerations, safety)	10-point Likert scale (1 = not important at all, 10 = very important)
Section V: Acceptability (2 items)	Acceptability was assessed at two levels: first, with regard to the integration of SARs into healthcare settings in general, and second, with respect to their use in professionals’ own clinical practice	4-point Likert scale (1 = very unfavorable, 2 = unfavorable, 3 = favorable, 4 = very favorable)

Regarding professional experience in healthcare, 37.4% of participants (*n* = 55/147) reported fewer than 5 years of experience, 29.9% (*n* = 44/147) reported between 5 and 15 years, and 32.7% (*n* = 48/147) reported more than 15 years.

#### 2.1.4 Workplace settings and patient populations

Regarding workplace settings, participants reported working primarily in hospital-based environments, with smaller proportions working in medico-social facilities (i.e., long-term care and social care institutions such as nursing homes or specialized residential facilities) and other contexts. Multiple responses were allowed to account for fragmented working time across settings. Overall, 81.8% of participants (*n* = 112/137) reported hospital-based work, 14.6% (*n* = 20/137) reported working in medico-social settings, 0.7% (*n* = 1/137) reported working in care coordination organizations, and 2.9% (*n* = 4/137) reported working in other settings.

A large majority of participants (90.3%, *n* = 131) reported working with older adults, while 37.2% (*n* = 54) reported working with adults and 17.9% (*n* = 26) with pediatric populations. One participant indicated working primarily with healthcare staff.

### 2.2 Measures

The study employed a structured questionnaire developed to assess healthcare professionals' familiarity with SARs, perceived usefulness across different SAR use cases, preferences regarding the type of robot they would be most inclined to use in healthcare, the perceived importance of implementation-related factors, and the acceptability of SARs in healthcare both in general settings and within respondents' own clinical practice, the latter being used as a proxy for their intention to use SARs in their everyday work. A study-specific instrument was used rather than an existing validated scale because the study aimed to capture context-specific use cases and implementation conditions for SARs in real-world healthcare settings, which are not fully addressed by currently available technology acceptance measures. The instrument's content and structure were informed by the existing literature on technology acceptance in healthcare and preliminary discussions with healthcare professionals to ensure contextual relevance.

The questionnaire included items assessing the perceived usefulness of robotic applications, as well as organizational, ethical, regulatory, and economic factors influencing their implementation in healthcare. These dimensions correspond to determinants described in implementation science frameworks such as the Consolidated Framework for Implementation Research (CFIR) (13), including intervention characteristics, the organizational context in which the intervention is implemented (inner setting), the broader external environment such as policies, regulations, and user needs (outer setting), characteristics of individuals (knowledge, beliefs, skills, and attitudes), and implementation processes.

Because the survey referred to different categories of robots, clear operational definitions were provided to respondents at the beginning of the questionnaire to standardize interpretation. Social robots were defined as devices used solely for social interaction (e.g., conversation, emotional support, companionship, or facilitation of social engagement), whereas assistive robots were defined as service-oriented systems (e.g., object manipulation, task reminders) that may also include social functions.

The questionnaire comprised five sections and 48 items. Response modalities included yes/no questions, open-ended fields, multiple-choice items, and Likert-type scales (1–10 for perceived usefulness and implementation-related factors; 1–4 for acceptability). An overview of the instrument and its sections is presented in Table 2.

**Table 2:** Overview of items and descriptions assessing perceived usefulness (by use case) and implementation-related factors for socially assistive robots.

Section	Short label	Description
Perceived usefulness of SARs by use case	Supporting learning	To support basic learning, education, or training activities for the user
	Psychological support	To help the user cope with stress and other psychological demands (comforting, helping them relax, reassuring, accompanying)
	Managing daily routine	To help the user manage daily routines (scheduling and task reminders)
	Assisting communication	To help the user communicate messages (written and verbal)
	Assisting videoconference communication	To help the user communicate via videoconferencing
	Assisting mobility	To assist the user with walking and mobility
	Assisting physical tasks	To help the user lift and carry objects
	Assisting feeding	To help the user eat and stay hydrated
	Therapeutic support	To provide therapeutic mediation (psychomotor therapy, speech therapy, psychotherapy)
	Helping health monitoring	To help monitor the person's condition (monitoring certain clinical parameters: activity, sleep, emotional state, etc.)
	Caregiver support	To assist family caregivers in their caregiving tasks
	Supporting social interaction	To facilitate the user's social interactions with others
	Recreational activities	Healthcare robotics to promote recreational and leisure activities
Types of robots participants would be most inclined to use	Facilitate patients’ social interactions	Robots intended to promote patients’ social interactions
	Support care provision	Robots to facilitate care provision (therapeutic mediation, rehabilitation, psychological support)
	Transport and delivery of equipment	Robots for the transport and delivery of equipment within healthcare settings
	Cleaning and disinfection	Robots for disinfection and cleaning in healthcare settings
	Telepresence communication	Robots for remote communication (videoconferencing)
SARs implementation-related factors	Defining health objectives	Identify a specific health objective for healthcare practices using SARs
	Identifying target populations	Identify the target population that could specifically benefit from healthcare practices using SARs
	Financial viability	Establishing financing and reimbursement pathways for SARs practices
	Demonstrating effectiveness	Scientific evaluation of the effectiveness of healthcare practices using SARs
	Clarifying stakeholder roles	Identification of key players who can provide healthcare practices using SARs
	Ensuring usability	Ensuring ease of use of SARs for patients and healthcare staff delivering the intervention
	Managing physical risks	Identification and prevention of physical risks associated with the use of SARs
	Managing psychological risks	Identification and prevention of psychological risks associated with the use of SARs
	Managing data-related risks	Identification and prevention of risks associated with the use of data by SARs
	Implementation costs	Assessing the costs of acquiring, installing, and maintaining SARs
	Cost–benefit balance	Conducting cost–benefit analyses of SAR-based practices
	Protecting vulnerable populations	Paying particular attention to the use of SARs with vulnerable populations
	Respecting user autonomy	Respecting the autonomy of the individual with regard to the use of SARs
	Preserving user dignity	Respecting the dignity of the individual when using SARs
	Ensuring equitable access	Ensuring equitable access to care practices using SARs
	Integration into care practices	Aligning SAR-based interventions with existing care practices
	Training healthcare professionals	Training professionals in SARs practices
	Employment impact	Assessing the impact of SAR implementation on employment in the health sector
	Professional acceptability	Accounting for variability in healthcare professionals' acceptability of SAR-based practices
	Patient and family acceptability	Considering patients’ and families’ acceptability of SAR-based practices
	Legal and regulatory frameworks	Ensuring legal compliance of healthcare practices using SARs

Sections III, IV of the questionnaire respectively focused on perceived usefulness across different clinical, psychosocial, and organizational use cases of SARs (e.g., patient support, logistics, agenda organization, and assistance to healthcare professionals) and preferred robot types, and on the assessment of implementation-related factors (including organizational, ethical, economic, technical, and legal dimensions). These categories were identified based on the existing literature in the field. A detailed overview of the corresponding items for these sections is provided in Supplementary Table 1.

As the questionnaire was not designed as a validated psychometric scale, a limited assessment of internal consistency was conducted. Cronbach's alpha indicated good internal consistency for the perceived usefulness items (*α* = 0.89) and excellent internal consistency for the implementation-related factors (*α* = 0.94). Item–rest correlations ranged from 0.44 to 0.73 and from 0.42 to 0.74, respectively.

#### 2.2.1 Data analysis

Data analyses were conducted using Jamovi software [version 2.4.11; (43)]. Analyses focused on four domains: sociodemographic and professional characteristics, perceived usefulness of SARs across use cases and preferences regarding the type of robot they would be most inclined to use in healthcare, perceived importance of implementation-related factors, acceptability of SARs in healthcare settings and in professionals own's clinical practice.

Descriptive statistics (frequencies, percentages, means, and standard deviations) were calculated for gender, age, professional category, years of experience, workplace setting, patient populations, and familiarity with SARs. Frequencies were also computed for the types of robots reported as known, encountered, or preferred in professional environments or clinical practice.

Perceived usefulness by use case and perceived importance of implementation-related factors were examined using descriptive statistics (means and standard deviations) and summarized graphically.

Acceptability of SARs was assessed at two levels: first, regarding their general use in healthcare, and second, with respect to their use in professionals’ own clinical practice. Differences across professional categories were examined using chi-square tests.

To assess the association between prior knowledge of SARs and acceptability, Mann–Whitney *U* tests were conducted to compare participants reporting prior knowledge of SARs with those without such knowledge, for both general healthcare use and clinical practice. Associations between prior knowledge of SARs and perceived importance of implementation-related factors were examined using nonparametric group comparisons.

A principal component analysis (PCA), followed by hierarchical clustering on principal components, was conducted to identify respondent profiles based on perceived usefulness by use case, implementation-related factors, and acceptability. Prior to conducting the principal component analysis (PCA), sampling adequacy was assessed using the Kaiser-Meyer-Olkin (KMO) measure and Bartlett's test of sphericity to verify the suitability of the correlation matrix for factor extraction. PCA was then performed on variables related to perceived usefulness, implementation-related factors, and acceptability. Component retention was guided by examination of eigenvalues and the scree plot.

To identify attitudinal profiles among respondents, hierarchical clustering on principal components (HCPC) was applied to the PCA results. The number of clusters was determined based on examination of cluster inertia and the interpretability of the resulting solution. Professional category was not included in the clustering procedure.

Associations between cluster membership and key variables were examined using chi-square tests of independence. Cluster structure was illustrated using a two-dimensional projection, with variables contributing to each dimension reported in Supplementary Tables 2a, b.

#### 2.2.2 Missing data and questionnaire completion

As is common in self-administered surveys, not all respondents completed the entire questionnaire. Among the 92 individuals who accessed the online version, 37 provided no data beyond the consent form (0% completion), 13 completed only the sociodemographic section (approximately 10% completion), and 5 completed the questionnaire up to the section on perceived usefulness by use case (approximately 75% completion). A total of 37 participants completed the full questionnaire online (100% completion). Because the different sections of the questionnaire were independent, only responses from participants who completed at least 75% of the questionnaire were retained for analysis, resulting in 42 online responses included in the final dataset. Analyses were performed using available-case analysis at the item level (no imputation), so the number of respondents may vary across specific tests.

For the paper-based version, 107 questionnaires were collected. One questionnaire contained only sociodemographic information (approximately 10% completion). The remaining 106 paper questionnaires met the inclusion criterion and were included in the analyses. The questionnaire completion flowchart is presented in Supplementary Figure 1.

## 3 Results

### 3.1 Prior knowledge and experience with socially assistive robots

Participants reported varying levels of prior knowledge of SARs. Table 3 summarizes the proportions of respondents who indicated knowledge of different robot categories, as well as those who reported exposure to robots in healthcare settings or in their own clinical practice.

**Table 3:** Distribution of healthcare professionals according to their prior knowledge and use of socially assistive robots.

Professional category	n (%)											
	Number of participant	Professional experience in healthcare (years)			Prior knowledge of social robots		Prior knowledge of assistive robots		Prior knowledge SARs use in healthcare settings		Previous use of SARs in their own clinical practice	
		<5	5–15	>15	Yes	No	Yes	No	Yes	No	Yes	No
Rehabilitation/reeducation	36 (25)	21 (58.3)	10 (27.8)	5 (13.9)	22 (61.1)	14 (38.9)	24 (66.7)	12 (33.3)	12 (33.3)	24 (66.7)	5 (13.9)	31 (86.1)
Care Assistance	25 (17.4)	2 (8)	14 (56)	9 (36)	16 (64)	9 (36)	11 (47.8)	12 (52.2)	9 (36)	16 (64)	3 (12)	22 (88)
Medical professions	19 (13.2)	2 (10.5)	8 (42.1)	9 (47.4)	12 (63.2)	7 (36.8)	14 (73.7)	5 (26.3)	7 (36.8)	12 (63.2)	3 (15.8)	16 (84.2)
Management/leadership	16 (11.1)	8 (53.3)	3 (20)	4 (26.7)	15 (93.7)	1 (6.3)	12 (80)	3 (20)	9 (56.2)	7 (43.8)	5 (31.2)	11 (68.8)
Nursing professions	12 (8.3)	1 (8.3)	3 (25)	8 (66.7)	7 (58.3)	5 (41.7)	6 (50)	6 (50)	4 (36.4)	7 (63.6)	0 (0)	12 (100)
Administrative/front-office	12 (8.3)	1 (8.3)	2 (16.7)	9 (75)	8 (66.7)	4 (33.3)	6 (50)	6 (50)	6 (50)	6 (50)	0 (0)	12 (100)
Students in health-related fields	9 (6.3)	9 (100)	0 (0)	0 (0)	5 (55.6)	4 (44.4)	5 (55.6)	4 (44.4)	1 (14.3)	6 (85.7)	1 (11.1)	8 (88.9)
Psychology	8 (5.6)	6 (75)	0 (0)	2 (25)	8 (100)	0 (0)	7 (87.5)	1 (12.5)	4 (50)	4 (50)	4 (50)	4 (50)
Dietitians	4 (2.8)	2 (50)	1 (25)	1 (25)	3 (75)	1 (25)	3 (75)	1 (25)	2 (50)	2 (50)	1 (25)	3 (75)
Social work	2 (1.4)	0 (0)	2 (100)	0 (0)	2 (100)	0 (0)	2 (100)	0 (0)	1 (50)	1 (50)	1 (50)	1 (50)
Total	143 (100)	55 (37.4)	44 (29.9)	48 (32.7)	100 (68)	48 (32)	94 (65)	51 (35)	55 (38)	90 (62)	23 (16)	125 (84)

**Figure 1:**
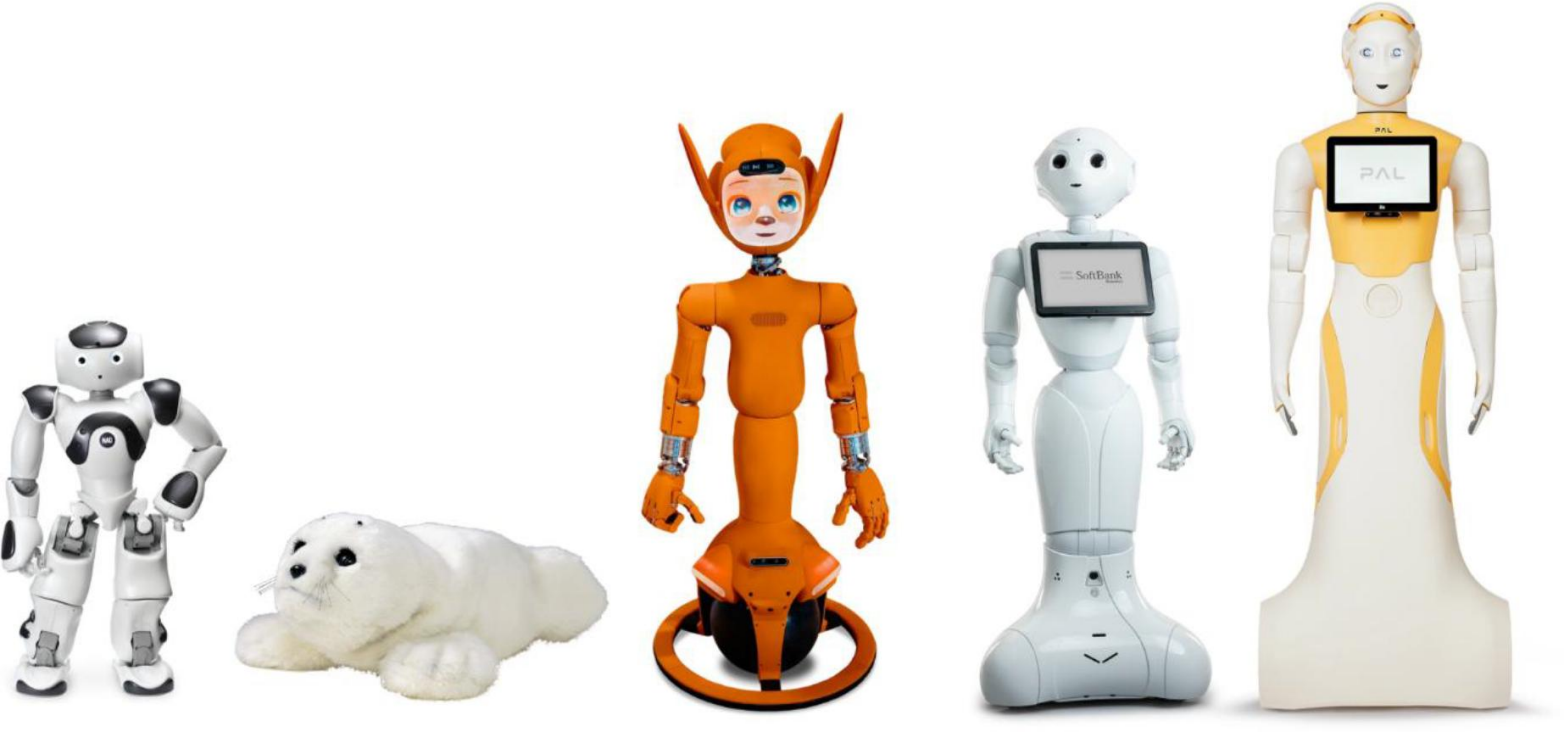
Most frequently mentioned socially assistive robots reported by participants. From left to right: Nao (Aldebaran), Paro (developed by Takanori Shibata), Mirokai (Enchanted Tools), Pepper (Aldebaran), and ARI (PAL Robotics).

Regarding prior knowledge, 68% (*n* = 100/148) reported being familiar with social robots, while 65% (*n* = 94/145) reported knowledge of assistive robots. Overall, 77% of participants (*n* = 114/148) reported having prior knowledge of either social robots, assistive robots, or both. Among these 114 participants, 37 cited specific robots used in their workplaces or professional practice. The most frequently mentioned robots were Nao and Paro (15 mentions each), followed by Mirokai (10 mentions), Pepper (9 mentions), and ARI (8 mentions). Robots such as Tiago (*n* = 3), UBBO (*n* = 2), CUTII (*n* = 2), and QT (*n* = 2) were mentioned less frequently. Several respondents (*n* = 10) referred to robots without naming a specific model (e.g., “robot for guidance,” “social robot for children,” “pet robot”); these responses were grouped under a non-identified robot category. Figure 1 illustrates the SARs most frequently mentioned by participants.

Regarding the use of SARs in the healthcare setting more generally or within respondents' own professional practice, 38% of participants (*n* = 55/148) reported being aware of SARs use in their professional environment, whereas only 16% (*n* = 23/148) reported current or prior personal use of SARs in their own practice.

### 3.2 Perceived usefulness per use case for socially assistive robots

Perceived usefulness of SARs across healthcare use cases was assessed on a 10-point scale ranging from 1 (very low usefulness) to 10 (very high usefulness). Mean scores ranged from 5.80 to 7.89, indicating overall positive evaluations across all domains. Based on predefined interpretative categories (1–3 = low usefulness; 4–5 = moderate usefulness; 6–7 = high usefulness; 8–10 = very high usefulness), no use case fell into the low usefulness range.

The highest perceived usefulness was observed for *assisting physical tasks* (M = 7.89, SD = 2.41), followed by *recreational activities* (M = 7.12, SD = 2.16), *managing daily routines* (M = 7.07, SD = 2.39), and *assisting communication* (M = 7.05, SD = 2.36), all within the high to very high range. Other domains such as *caregiver support*, *videoconferencing, health monitoring*, *mobility*, *learning*, *social interaction*, and *psychological support* also achieved mean scores in the high usefulness range (6–7). Only two use cases were rated in the “moderate usefulness” range: *therapeutic support* (M = 5.86, SD = 2.77) and *assisting feeding* (M = 5.80, SD = 2.74), suggesting greater caution regarding these applications.

**Figure 2:**
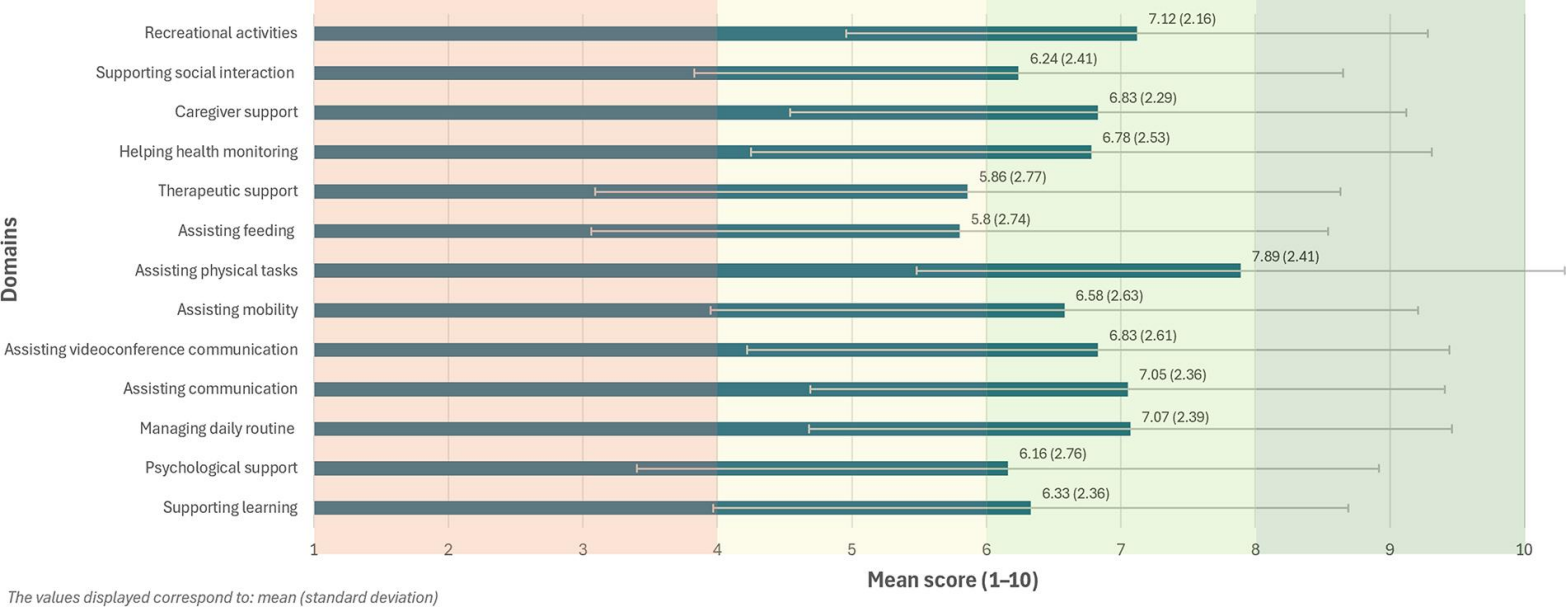
Perceived usefulness of SARs use cases in healthcare practice.

Figure 2 presents the distribution of mean usefulness ratings and their variability across all use cases.

#### 3.2.1 Preferences regarding the types of robots healthcare professionals would be most inclined to use

Among the 148 participants, 134 answered the multiple-response question regarding the types of robots they would be most inclined to use. The most frequently cited type of robot was for transport and delivery of equipment (57%), followed by robots for cleaning and disinfection (53%). Robots intended to facilitate patients' social interactions (45%) and to support care provision (45%) were mentioned by a similar proportion of participants, while telepresence robots were cited by 43% of respondents.

### 3.3 Perceived importance of implementation-related factors for socially assistive robots

Perceived importance of implementation-related factors was assessed on a 10-point scale ranging from 1 (not important at all) to 10 (very important). Mean scores ranged from 6.78 to 8.34, indicating that all factors were judged to have at least high importance. No factor was rated in the “low” or “moderate importance” ranges.

Factors rated as having “very high importance” primarily concerned ethical, legal, and practical safeguards. These included defining a *legal and regulatory framework* (M = 8.34, SD = 2.15), *preserving user dignity* (M = 8.23, SD = 2.56), *training healthcare professionals* (M = 8.14, SD = 2.09), *ensuring patient acceptability* (M = 8.11, SD = 2.17), *respecting user autonomy* (M = 8.05, SD = 2.28), and *ensuring ease of use* (M = 7.99, SD = 2.10, bordering the very high range).

The remaining factors, including *management of physical*, *psychological, and data-related risks*; *vigilance for vulnerable populations*; *equitable access*; *cost–benefit assessment*; *scientific evidence of effectiveness*; *identification of target populations*; *integration into existing care practices*; *funding mechanisms*; *clarification of stakeholders' roles*; and *employment impacts*, were all rated in the “high importance” range, underscoring that professionals view these elements as necessary conditions for SAR implementation rather than optional considerations

**Figure 3:**
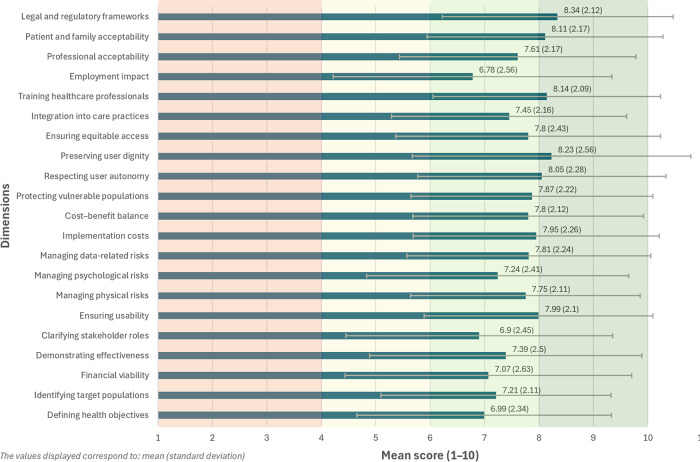
Implementation-related factors of SARs-based healthcare practices.

Figure 3 presents the perceived importance of implementation-related factors for SARs and illustrates mean importance ratings and their variability across dimensions.

### 3.4 Acceptability of socially assistive robots in healthcare settings and professionals' own clinical practice

**Figure 4:**
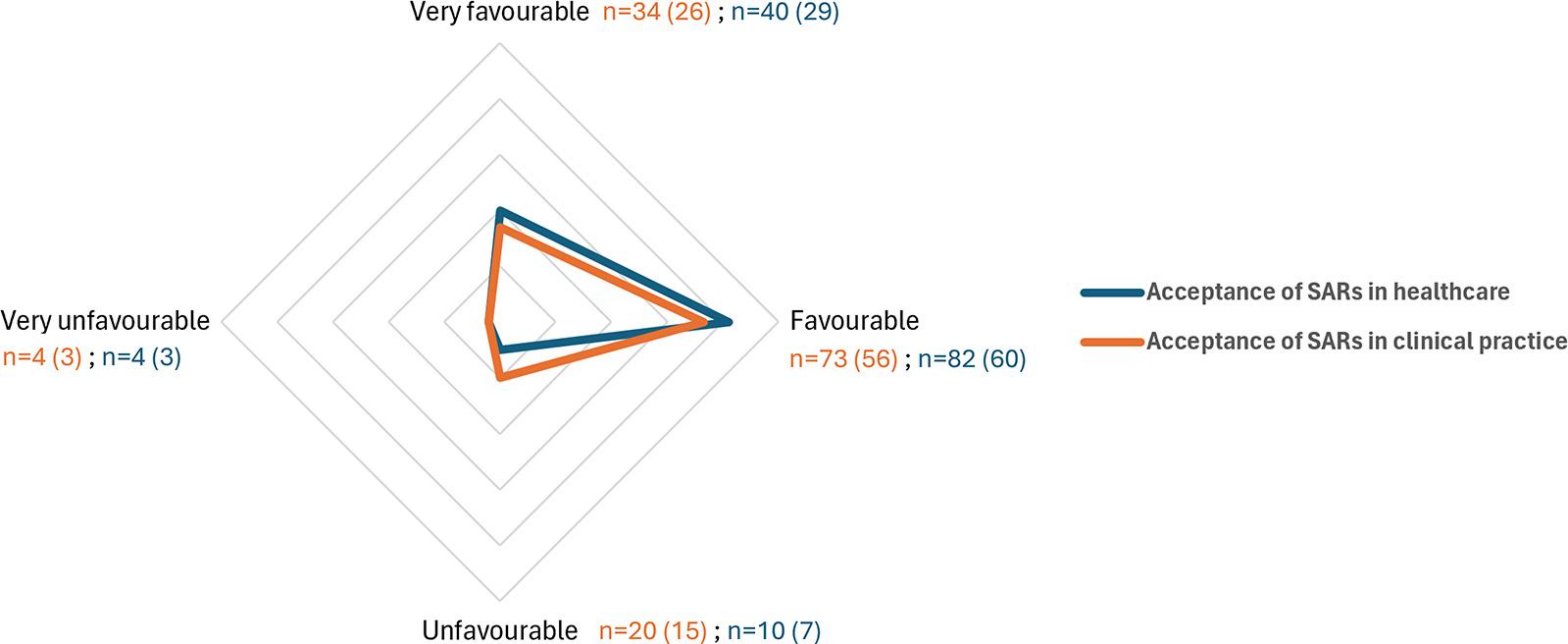
Healthcare professionals’ acceptability of SARs in healthcare settings and in their own clinical practice.

Participants generally expressed a positive attitude toward the use of SARs. With regard to the general use of SARs in healthcare settings, 89% of respondents (*n* = 136) reported being favourable or very favourable (60% favourable; 29% very favourable). Acceptability ratings were slightly lower when participants were asked to consider use in their own clinical practice (proxy for intention to use), with 82% expressing a favourable or very favourable opinion (*n* = 137; 56% favourable; 26% very favourable). Figure 4 summarizes the distribution of acceptability ratings for both general healthcare settings and professionals' own clinical practice.

### 3.5 Acceptability of socially assistive robots in healthcare by professional group

#### 3.5.1 Healthcare professionals' acceptability of socially assistive robots integration into healthcare settings

Although the effect of professional category on acceptability of SARs in healthcare settings was statistically significant [*χ*^2^(9, *N* = 136) = 17.13, *p* = .047], *post hoc* Dwass–Steel–Critchlow–Fligner tests with correction did not reveal any significant pairwise differences between professional groups (all *p* > .05). Nevertheless, inspection of response distributions revealed marked contrasts across professions.

Social work professionals (*n* = 2, M = 4.00/4, SD = 0) reported exclusively very favorable attitudes, corresponding to the highest level of acceptability. Care assistants (*n* = 24, M = 3.17/4, SD = 0.64) and administrative/front-office staff (*n* = 7, M = 3.14/4, SD = 0.69) also displayed a high proportion of favorable responses. Nurses (*n* = 10, M = 3.00/4, SD = 0.67) and psychologists (*n* = 7, M = 3.00/4, SD = 1.00) showed more intermediate profiles. In contrast, rehabilitation and re-education professionals (*n* = 33, M = 2.73/4, SD = 0.72) exhibited the highest proportion of unfavorable responses, constituting the professional group least supportive of the integration of socially assistive robots into general healthcare settings. Figure 4 provides a detailed visualization of acceptability distributions across professional groups.

#### 3.5.2 Acceptability with respect to use in professionals' own clinical practice

Acceptability of using SARs within professionals' own clinical practice did not differ significantly across professional categories [*χ*^2^(9, *N* = 131) = 15.18, *p* = .086]. The highest mean acceptability scores were observed among social work professionals (*n* = 2, M = 4.00/4, SD = 0), professionals in management and leadership roles (*n* = 14, M = 3.29/4, SD = 0.47), and medical professionals (*n* = 18, M = 3.28/4, SD = 0.67). In contrast, rehabilitation and re-education professionals reported the lowest mean acceptability (*n* = 35, M = 2.80/4, SD = 0.80). Mean acceptability scores for the remaining professional groups fell within a generally favorable range, between 3.10/4 (*n* = 10, SD = 0.57) and 3.43/4 (*n* = 7, SD = 0.53).

### 3.6 Effects of prior knowledge and/or use of socially assistive robots on acceptability

#### 3.6.1 Association between prior knowledge of social assistive robots and acceptability in healthcare settings

A Mann–Whitney *U* test revealed a statistically significant difference in acceptability regarding the integration of SARs into general healthcare settings between participants with prior knowledge of SARs and those without such knowledge (*U* = 1230, *p* = .030, *r* = .23). Participants reporting prior knowledge of SARs expressed higher levels of acceptability.

In contrast, no statistically significant difference was observed for acceptability with respect to the use of SARs in professionals' own clinical practice between participants with and without prior knowledge of SARs (*U* = 1,178, *p* = .064).

#### 3.6.2 Acceptability of robots according to professional exposure and personal use

A Mann–Whitney *U* test revealed no statistically significant difference in acceptability regarding the integration of robots into healthcare settings between participants who reported being aware of robot use in their professional environment and those who did not, either for acceptability in their own professional practice (*U* = 1,595.5, *p* = .076) or into general healthcare settings acceptability (*U* = 1,823.5, *p* = .180).

In contrast, a statistically significant difference in acceptability between participants who reported current or prior personal use of robots in their professional practice and those who did not. Participants reporting personal use expressed significantly lower acceptability, both regarding acceptability in their own professional practice (*U* = 725.0, *p* = .003) and general acceptability in healthcare settings (*U* = 809.5, *p* = .006).

#### 3.6.3 Prior knowledge of socially assistive robots and perceived importance of implementation-related factors

Participants with prior knowledge of SARs attributed significantly greater importance to several key implementation-related factors compared with those without prior knowledge, particularly in ethical and normative domains. The largest differences were observed for preserving user dignity (M = 8.69 vs. 6.67, *p* < .001, r = .84), respecting user autonomy (M = 8.44 vs. 6.70, *p* < .001, r = .80), and protecting vulnerable populations (M = 8.25 vs. 6.58, *p* < .001, *r* = .79), all associated with large effect sizes (Table 4).

**Table 4:** Acceptability of using socially assistive robots in professionals’ own clinical practice by professional category.

Professional categories	Very unfavorable *n* (%)	Unfavorable *n* (%)	Favorable *n* (%)	Very favorable *n* (%)
Rehabilitation/reeducation	1 (3.03)	11 (33.33)	17 (51.52)	4 (12.12)
Care Assistance	0 (0)	3 (12.5)	14 (58.33)	7 (29.17)
Medical professions	0 (0)	2 (11.11)	8 (50)	7 (38.89)
Management/leadership	0 (0)	0 (0)	10 (71.43)	4 (28.57)
Nursing professions	0 (0)	2 (20)	6 (60)	2 (20)
Administrative/front-office	0 (0)	1 (14.29)	4 (57.14)	2 (28.57)
Students in health-related fields	0 (0)	1 (11.11)	6 (66.67)	2 (22.22)
Psychology	1 (14.29)	0 (0)	4 (57.14)	2 (28.57)
Dietitians	1 (33.33)	0 (0)	1 (33.33)	1 (33.33)
Social work	0 (0)	0 (0)	0 (0)	2 (100)
Total	3 (2.32)	20 (15.38)	73 (56.15)	34 (26.15)

Substantial differences were also found for factors related to access and governance, including ensuring equitable access to SAR-based practices (M = 8.11 vs. 6.77, *p* = .007, *r* = .56) and defining a legal and regulatory framework (M = 8.56 vs. 7.60, *p* = .029, *r* = .46). Overall, prior knowledge of SARs was associated with a stronger emphasis on ethical safeguards and structural conditions for implementation.

#### 3.6.4 Prior knowledge of socially assistive robots and perceived usefulness across use cases

Prior knowledge of SARs was also associated with higher perceived usefulness for several psychosocial and support-oriented use cases. As shown in Table 5, participants with prior knowledge of SARs rated SARs as significantly more useful for *supporting social interactions* (M = 6.54 vs. 5.26, *p* = .007, *r* = .54), *therapeutic support* (M = 6.15 vs. 4.88, *p* = .022, *r* = .47), and *caregiver support* (M = 7.06 vs. 6.00, *p* = .020, *r* = .47) compared with participants without prior knowledge. These differences were associated with moderate effect sizes, indicating that familiarity with SARs was linked to more favorable evaluations of SARs potential usefulness. Table 6 summarizes healthcare professionals' overall perceptions of the usefulness and acceptability of socially assistive robots across the different use cases considered in the survey.

**Table 5:** Effect of prior knowledge on implementation-related factors.

Implementation-related factor domain	Prior knowledge Mean (SD)	No prior knowledge Mean (SD)	Statistic (df)	p-value	Effect size
Identifying target population	7.50 (1.98)	6.19 (2.24)	3.15 (138)	.002	.64
Demonstrating effectiveness	7.69 (2.40)	6.38 (2.64)	2.67 (140)	.008	.54
Ensuring usability	8.23 (1.83)	7.25 (2.69)	2.33 (130)	.021	.47
Managing physical risk	8.00 (1.87)	6.97 (2.62)	2.45 (131)	.016	.50
Protecting vulnerable populations	8.25 (1.88)	6.58 (2.75)	3.86 (133)	<.001	.79
Respecting user autonomy	8.44 (1.99)	6.70 (2.71)	3.87 (132)	<.001	.80
Preserving user dignity	8.69 (2.16)	6.67 (3.18)	4.03 (131)	<.001	.84
Ensuring equitable access	8.11 (2.24)	6.77 (2.80)	2.72 (130)	.007	.56
Employment impact	7.74 (2.00)	6.50 (2.45)	2.82 (130)	.006	.59
Legal and regulatory framework	8.56 (1.90)	7.60 (2.65)	2.21 (132)	.029	.46

**Table 6:** Effect of prior knowledge on perceived usefulness of socially assistive robots use cases in healthcare.

Perceived usefulness per use case domain	Prior knowledge Mean (SD)	No prior knowledge Mean (SD)	Statistic (df)	p-value	Effect size
Therapeutic support	6.15 (2.69)	4.88 (2.85)	2.32 (140)	.022	.47
Caregiver support	7.06 (2.09)	6.00 (2.74)	2.35 (141)	.020	.47
Supporting social interactions	6.54 (2.24)	5.26 (2.69)	2.75 (142)	.007	.54

### 3.7 Cluster profiles of attitudes toward socially assistive robots

**Figure 5:**
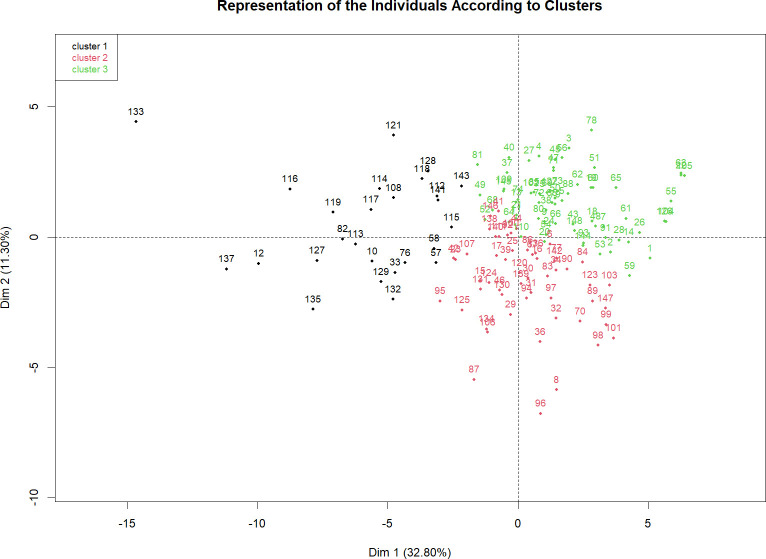
Representation of the individuals according to clusters (black: low acceptability group, red: moderate acceptability group, green: high acceptability group).

A PCA followed by HCPC was conducted to identify distinct respondent profiles based on similarities in individual response patterns across perceived usefulness by use case, implementation-related factors, and acceptability. Professional category and sociodemographic variables were not included as clustering variables. The KMO measure indicated acceptable sampling adequacy (KMO = 0.76), and Bartlett's test of sphericity was significant [*χ*^2^(630) = 2,372.95, *p* < .001]. Individual KMO values ranged from 0.59 to 0.87. Examination of the eigenvalues and cumulative explained variance (Supplementary Table 2) indicated that a three-cluster solution (Figure 5) provided the most interpretable and stable representation of response patterns.

No predefined cut-off values were applied to acceptability scores; instead, clusters were labelled “low,” “moderate,” and “high” acceptability based on their relative positions across acceptability-related dimensions. Cluster 1 was characterized by mean acceptability scores in the lower range of the observed distribution (<5/10), Cluster 2 by intermediate values (<7/10), and Cluster 3 by high mean scores (≥8/10). The three clusters differed in overall acceptability, perceived usefulness across healthcare use cases, and the importance attributed to implementation-related factors. Table 7 presents the sociodemographic and professional characteristics of participants within each cluster.

**Table 7:** Socio-demographic and professional characteristics of clusters.

Cluster		Cluster 1 Low acceptability (*N* = 26)	Cluster 2 Moderate acceptability (*N* = 54)	Cluster 3 High acceptability (*N* = 68)
Gender *N* (%)	Female	21 (80.8%)	40 (74.1%)	51 (75.0%)
	Man	4 (15.4%)	14 (25.9%)	17 (25.0%)
	Other	1 (3.8%)	0 (0%)	0 (0%)
Age, Mean (SD), Min-Max		39.5 (14.4) 20–67	32.6 (11.4) 18–66	42.5 (13.9) 19–78
Professions *N* (%)	Medical professions	1 (3.8%)	2 (3.7%)	3 (4.4%)
	Psychology	3 (11.5%)	6 (11.1%)	10 (14.7%)
	Nursing professions	2 (7.7%)	2 (3.7%)	8 (11.8%)
	Students in health-related fields	0 (0%)	6 (11.1%)	3 (4.4%)
	Management/leadership	1 (3.8%)	4 (7.4%)	11 (16.2%)
	Administrative/front-office	0 (0%)	2 (3.7%)	10 (14.7%)
	Dietitians	0 (0%)	1 (1.9%)	2 (4.4%)
	Social work	0 (0%)	0 (0%)	2 (2.9%)
	Care Assistance	7 (26.9%)	9 (16.7%)	9 (13.2%)
	Rehabilitation/reeducation	12 (46.2%)	18 (33.3%)	6 (8.8%)
Professional experience *N* (%)	Less than 5 years	11 (42.3%)	26 (48.1%)	18 (26.5%)
	Between 5 and 15 years	8 (30.8%)	18 (33.3%)	18 (26.2%)
	Over 15 years	7 (26.9%)	10 (18.5%)	31 (45.6%)

On this basis, three distinct attitudinal profiles emerged, corresponding to clusters with low, moderate, and high levels of acceptability, which are described in detail below in terms of prior exposure to SARs, perceived usefulness, importance attributed to implementation-related factors, and professional characteristics.

#### 3.7.1 Cluster 1—low-acceptability profile (*n* = 26)

- *Knowledge/exposure:* Cluster 1 contained the highest proportion of participants without prior knowledge of SARs, with half of respondents reporting no previous exposure (50%, *n* = 13/26).
- *Ratings:* This group displayed consistently lower ratings of perceived usefulness across use cases and attributed lower importance to implementation-related factors compared with the other clusters. Acceptability ratings were predominantly in the unfavorable range, both for the integration of SARs into healthcare in general and for their use in one's own clinical practice.
- *Professional composition and experience:* Professionally, Cluster 1 included a high representation of rehabilitation and re-education professionals (46.2%) and care assistance staff (26.9%), with few participants in managerial or administrative roles. Participants tended to have shorter professional experience, with 42.3% reporting fewer than five years in healthcare practice.
- *Interpretive label:* Overall, Cluster 1 reflects a profile of limited familiarity with SARs, low perceived usefulness, and low acceptability, combined with relatively low importance attached to implementation conditions, a “low-acceptability, low-exposure” profile rather than active opposition.

#### 3.7.2 Cluster 2—moderate-acceptability profile (*n* = 54)

- *Knowledge/exposure:* A substantial majority of participants in Cluster 2 reported prior knowledge of SARs (77.8%, *n* = 42/54).
- *Ratings:* Participants in this cluster generally reported moderate to favorable acceptability, accompanied by intermediate ratings of perceived usefulness across use cases. Importantly, they attributed relatively high importance to implementation-related factors, sometimes comparable to or higher than Cluster 3 for certain dimensions, indicating heightened attention to conditions required for appropriate deployment.
- *Professional composition and experience:* Cluster 2 showed a diverse professional composition, including rehabilitation professionals (33.3%), care assistance staff (16.7%), students in health-related fields (11.1%), and management or leadership roles (7.4%). In terms of experience, this cluster was skewed toward earlier career stages, with nearly half of participants reporting fewer than five years of professional experience (48.1%).
- *Interpretive label:* Cluster 2 can be interpreted as a “moderate-acceptability, cautious-but-open” profile, characterized by openness to SARs combined with strong attention to implementation requirements.

#### 3.7.3 Cluster 3—high-acceptability profile (*n* = 68)

- *Knowledge/exposure:* Cluster 3 included the highest proportion of participants with prior knowledge of SARs (86.8%, *n* = 59/68).
- *Ratings:* This group was defined by consistently high acceptability ratings and high perceived usefulness across most healthcare use cases, including daily care organization, mobility assistance, communication support, and health monitoring. Participants also attributed high importance to implementation-related factors, indicating that strong acceptability co-exists with careful consideration of ethical, organizational, legal, and practical conditions.
- *Professional composition and experience:* Compared with the other clusters, Cluster 3 comprised a larger share of participants with extensive professional experience, with 45.6% reporting more than 15 years in healthcare practice. Administrative/front-office staff (14.7%) and management/leadership roles (16.2%) were more prevalent in this group, whereas rehabilitation professionals were less represented (8.8%).
- *Interpretive label:* Overall, Cluster 3 corresponds to a “high-acceptability, experienced” profile, characterized by broad perceived usefulness, strong importance attributed to implementation conditions, and high acceptability of SAR use in healthcare settings in general.

#### 3.7.4 Visualization of the three attitudinal clusters in principal component analysis space

To visually illustrate the distribution of participants across clusters, a two-dimensional scatterplot projection was generated based on the first two principal components (Figure 5). Each point represents an individual participant, with colors indicating cluster membership (low = black; moderate = red;, and high = green acceptability profiles). The variables contributing to each dimension, along with their respective weights and explained variance, are reported in Supplementary Tables 1a, b.

Figure 5 highlights the spatial separation of the three clusters within the factorial space, reflecting distinct response patterns across perceived usefulness by use case, importance attributed to implementation-related factors, and acceptability. The projection illustrates how the three clusters occupy different regions of the multidimensional space, consistent with the attitudinal profiles described above, and shows the relative overlap and dispersion of participants within each group.

The two dimensions displayed in Figure 5 correspond to the first two principal components derived from the principal component analysis. These components represent linear combinations of the original survey variables and summarize the main patterns of variance in participants' responses across perceived usefulness by use case, importance attributed to implementation-related factors, and acceptability. Dimension 1 accounted for 32.8% of the total variance, and Dimension 2 accounted for 11.3%.

**Figure 6:**
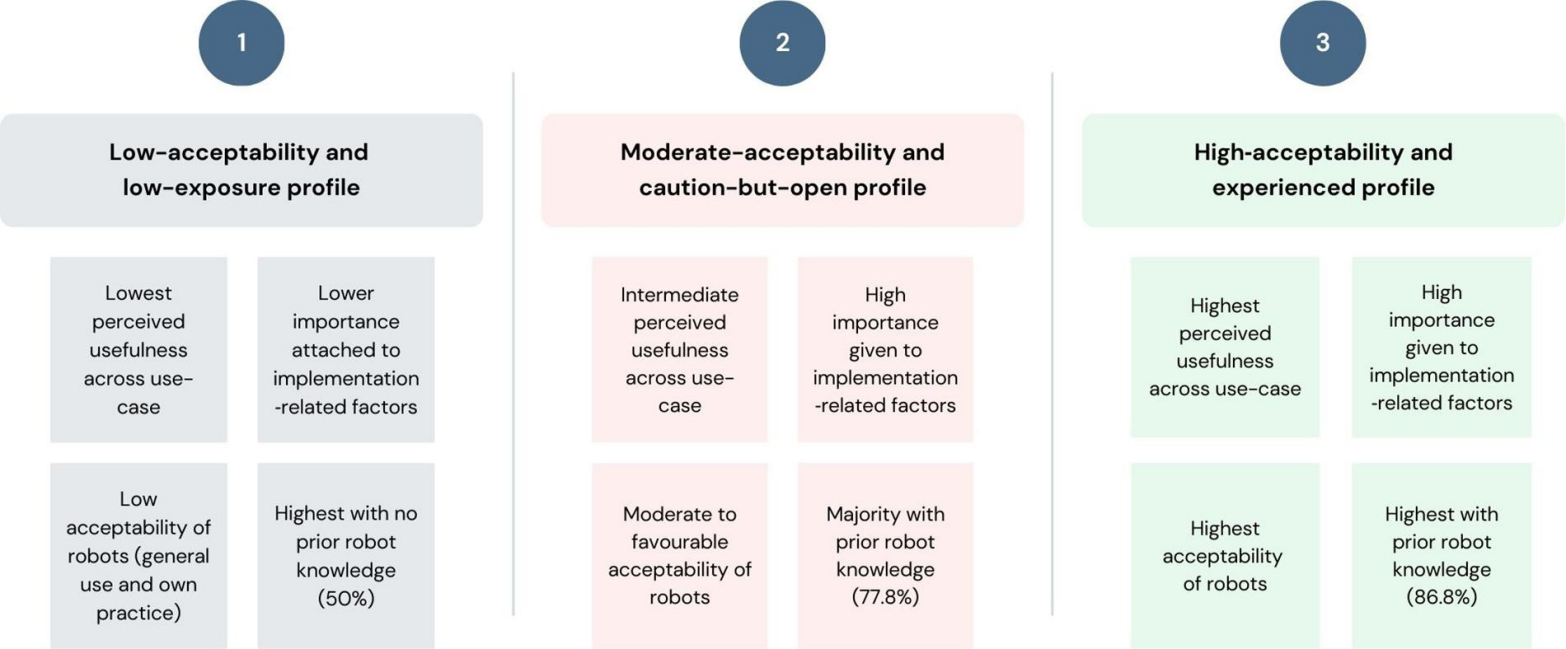
Profiles of healthcare professionals based on attitudes toward socially assistive robots.

Figure 6 presents a synthetic representation of the three attitudinal profiles identified through the cluster analysis, summarizing key patterns of perceived usefulness, importance attributed to implementation-related factors, acceptability, and prior knowledge of SARs for each cluster. Table 7 presents the sociodemographic and professional characteristics of participants within each cluster.

### 3.8 Associations between cluster membership, sociodemographic characteristics, and prior knowledge of socially assistive robots

Chi-square tests were conducted to examine associations between cluster membership and selected sociodemographic variables, as well as prior knowledge of SARs. As expected, cluster membership was strongly associated with acceptability of SARs in general healthcare settings [*χ*^2^(6, *N* = 148) = 52.0, *p* < .001]. A similarly strong association was observed for acceptability regarding the use of SARs in professionals' own clinical practice [*χ*^2^(6, *N* = 148) = 46.0, *p* < .001].

Prior knowledge of SARs differed significantly across clusters [*χ*^2^(2, *N* = 148) = 14.0, *p* < .001], with higher proportions of participants reporting prior knowledge in the moderate- and high-acceptability clusters (Clusters 2 and 3). In addition, professional category was significantly associated with cluster membership [*χ*^2^(18, *N* = 148) = 37.0, *p* = .005], as was level of professional experience [*χ*^2^(4, *N* = 148) = 12.0, *p* = .020]. In contrast, gender was not significantly associated with cluster membership [*χ*^2^(4, *N* = 148) = 5.7, *p* = .20].

## 4 Discussion

### 4.1 Synthesis of results

This study examined healthcare professionals' perceptions and acceptability of SARs, as well as attitudinal profiles toward their use, based on self-reported data collected across diverse professional roles and care settings. The findings should be interpreted as exploratory insights into how healthcare professionals currently perceive SARs and the conditions they consider relevant for their potential integration into clinical and care environments. Overall, the results indicate generally favorable attitudes toward SARs, alongside differentiated evaluations of perceived usefulness across use cases, strong attention to ethical and organizational conditions for implementation, and the identification of distinct profiles of acceptance. Together, these results suggest that professionals' views of SARs are structured, multidimensional, and contingent on both perceived functional relevance and contextual implementation requirements.

### 4.2 Perceived usefulness across use cases

Healthcare professionals in this study generally perceived SAR use cases as moderately to highly useful, with clear preferences across domains.

In contrast, use cases involving therapeutic and psychosocial care were evaluated more cautiously. Therapeutic support and assisting feeding were the only applications rated within the moderate usefulness range, while other psychosocial uses (including psychological support and supporting social interaction) received lower ratings than more functional or organizational tasks. Notably, no use case was rated in the low usefulness range, suggesting that participants saw at least some potential value in all proposed applications.

This pattern aligns with previous research showing that healthcare professionals tend to value SARs primarily for practical, supportive, and non-intimate functions. Turja et al. (26), reported higher acceptance of SARs for physical and logistical tasks than for functions involving the relational or interactional core of care. Similarly, the systematic review by Trainum et al. (27) highlights that professional acceptance of SARs is closely linked to their perceived contribution to workload reduction and support for everyday activities, rather than to roles requiring therapeutic judgment or sustained emotional engagement.

Qualitative studies further support this selective pattern of acceptance. Papadopoulos et al. (44) showed that caregivers primarily envision SARs as tools for practical assistance, while expressing reservations regarding their involvement in emotionally demanding situations, or complex interactions. Rigaud et al. (45) similarly reported that professionals perceive robots as particularly appropriate for non-intimate and supportive uses (such as telepresence, recreational activities, cognitive stimulation, and object retrieval). Vänni and Salin (46) further emphasized their potential to enhance productivityand support care activities without replacing human relationships.

The present study shows that prior knowledge of SARs is associated with broader perceptions of usefulness, particularly for psychosocial applications (including *supporting social interaction*, *therapeutic support*, and *caregiver support*). This suggests that familiarity with SARs, may expand the range of use cases professionals consider relevant.

### 4.3 Implementation-related factors are viewed as necessary conditions for socially assistive robots integration

Healthcare professionals in this study attributed “high” to “very high importance” to all implementation-related factors related to SAR-based healthcare practices. Factors rated as “very high importance” primarily concerned ethical and legal safeguards and operational and professional readiness conditions. priorities reflect professionals' emphasis on protecting patients' rights and ensuring that SAR use remains aligned with fundamental ethical principles and regulatory obligations. This pattern is consistent with previous literature highlighting the central role of ethical and legal considerations in professionals' evaluations of SARs. For example, Lee et al. (29) identify legal compliance, data protection, safety, and equity of access as key determinants of professional perspectives. Other studies also highlighted ethical risks to autonomy and dignity when robots were used to monitor, control, or replace human interaction (10, 20, 21, 47).

*Training healthcare professionals* and *ensuring ease of use* were also rated as very important, highlighting the need for professional and operational readiness for safe SARs use. This finding aligns with previous research indicating that insufficient knowledge of SARs capabilities constitutes a major barrier, while structured training supports acceptance and sustained use (38, 39, 48).

A second group of factors, rated within the “high importance” range, related to risk prevention, protection of vulnerable populations, and feasibility of use in practice. This reflects a precautionary approach in which SARs potential benefits are systematically assessed alongside risks, particularly for populations requiring increased protection. Similar concerns are highlighted in the review by Lee et al. (29), which emphasizes safety, effectiveness, and equity of access as key determinants of professional evaluations of SARs.

Finally, organizational and system-level considerations, received slightly lower, though still high, importance ratings. This pattern may reflect professionals' stronger sensitivity to conditions directly affecting everyday practice, whereas more strategic governance issues may appear less immediate. This interpretation is supported by Lee et al. (29), who note that perceived usefulness, ease of use, and training tend to be foregrounded in professional evaluations, whereas broader organizational reconfigurations often remain less salient. Similarly, Leoste et al. (47) observed that concrete training and use scenarios increased professionals' confidence and willingness to integrate a robot into care routines, while questions related to team roles, task distribution, and longer-term organizational implications remained more open.

The present study also shows that prior knowledge of SARs is associated with attributing greater importance to a wider range of implementation-related factors. Participants familiar with SARs rated dimensions such as dignity, autonomy, equity of access, safety, integration into practice, and the legal framework as more important than those without prior knowledge. This suggests that familiarity is associated not simply with higher acceptance, but with more differentiated and demanding evaluations of implementation conditions. Similar patterns have been reported for other healthcare technologies, where experience increases awareness of constraints alongside perceived benefits (28).

### 4.4 Acceptability of SARs: favorable attitudes influenced by knowledge, experience, and context

In this study, acceptability was examined at two distinct levels: acceptability of SARs in healthcare settings in general, reflecting system-level evaluations, and acceptability of SARs in one's own clinical practice, which was used as a proxy for professionals' intention to use SARs in their everyday work. Overall, healthcare professionals expressed favorable to very favorable attitudes toward SAR integration at both levels. A large majority of participants reported being “favorable” or “very favorable” toward SAR use in healthcare practice in general, while acceptability, and thus intention to use, was slightly lower when participants were asked to consider use within their own clinical practice. This systematic difference suggests that general approval of SARs does not automatically translate into readiness for personal adoption in everyday professional activities. Similar distinctions between global attitudes and individual intention to use have been reported in previous research, in which healthcare professionals and students expressed positive views toward robots while reporting more limited willingness to integrate them into their own practice (49, 50).

Beyond this global trend, acceptability was influenced by different forms of familiarity and exposure to SARs. Participants reporting prior knowledge of SARs expressed significantly higher acceptability regarding their integration into healthcare settings in general, suggesting that familiarity may support positive attitudes. In contrast, prior knowledge did not significantly influence acceptability when participants considered the use of SARs within their own clinical practice. This dissociation indicates that conceptual or indirect familiarity may facilitate general acceptance, while personal adoption decisions and intentions to use remain influenced by additional factors related to feasibility, responsibility, training, and integration into routine care.

Importantly, awareness of SAR use within one's professional environment (i.e., knowing that SARs are used in related healthcare settings) was not associated with acceptability, either at the general level or in relation to one's own clinical practice. By contrast, participants who reported current or prior personal use of SARs in their professional practice expressed significantly *lower* acceptability, both regarding use in their own practice and SAR integration in healthcare settings more broadly. This finding suggests that direct experience does not necessarily reinforce initial enthusiasm and may instead lead to more critical or cautious evaluations.

This pattern partly converges with, but also extends, findings from an Italian survey by Carradore et al. (41), which reported generally positive expectations regarding the potential of SARs in healthcare despite limited direct experience among respondents. While that study emphasized optimism in contexts where exposure remained limited, the present findings indicate that greater proximity to real use situations may temper expectations, resulting in more differentiated evaluations. In this sense, the two studies are not contradictory but rather highlight different points along a continuum of exposure, from prospective acceptance to experience-based appraisal.

These results can be further interpreted in light of broader methodological critiques of acceptability research in social robotics. David et al. (51) underline that, despite a growing number of studies, most assessments of SARs acceptability are conducted in the short term and under constrained or artificial conditions, while genuinely long-term and ecological studies remain scarce. Evidence from the few longitudinal studies conducted in real-life contexts suggests that acceptability is neither stable nor unidirectional. For example, longitudinal home studies of domestic social robots by de Graaf et al. (52), show that evaluations evolve over time: for some users, repeated interaction and familiarity lead to increased acceptability as concrete benefits and everyday uses are discovered; for others, growing awareness of limited utility, errors, or functional shortcomings results in declining enthusiasm, revised expectations, and even discontinuation of use.

In this context, the lower acceptability reported by participants with direct experience of SAR use in the present study should not be interpreted as resistance to the technology, but rather as reflecting more realistic and experience-based judgments compared with participants without such experience, even though overall acceptability levels remained high in absolute terms. This suggests that acceptability of SARs may increase or decrease depending on how perceived benefits, constraints, and organizational conditions are encountered over time, rather than following a single linear trajectory. This reinforces the interpretation of acceptability as a dynamic and context-dependent construct and suggests that cross-sectional survey measures capture only a snapshot within an evolving process of evaluation and appropriation.

Finally, descriptive analyses revealed variability in acceptability across professional categories, with rehabilitation professionals tending to report lower acceptability and social work and care-assistance professionals higher levels. However, *post hoc* analyses did not identify statistically significant pairwise differences between professional groups. Given the heterogeneous composition of the sample and the wide variability in prior knowledge and experience across professions, these findings suggest that professional category alone does not adequately explain acceptability patterns, which is consistent with previous findings (17).

### 4.5 Attitudinal profiles highlight heterogeneity in acceptance patterns

Cluster analysis identified three distinct attitudinal profiles toward SARs: a low-acceptability profile, a moderate-acceptability profile, and a high-acceptability profile. The *low-acceptability* cluster was characterized by consistently low ratings across perceived usefulness by use case and importance attributed to implementation-related factors and included the highest proportion of participants without prior knowledge of SARs. In contrast, the *high-acceptability* cluster showed uniformly high ratings across all dimensions and included a large proportion of participants reporting prior knowledge of SARs. The *moderate-acceptability* cluster combined generally favorable attitudes toward SARs with intermediate evaluations of perceived usefulness by use case and heightened attention to implementation-related factors.

Similar patterns have been reported in previous studies identifying heterogeneous attitudinal profiles toward SARs, typically distinguishing between low-acceptability groups, moderately supportive but cautious profiles, and high acceptability profiles (40). Prior research suggests that such profiles are less strongly associated with professional role or sociodemographic characteristics than with familiarity with SARs, expectations regarding perceived usefulness, and ethical or organizational considerations (33, 53). In line with these findings, cluster membership in the present study was not significantly associated with profession, gender, or years of professional experience, but was strongly related to acceptability measures and prior knowledge of SARs.

This result suggests that differences in attitudes toward SARs is primarily associated with perceptual and experiential factors, rather than with sociodemographic or professional characteristics contrary to Bi et al. (40) study in which they found that demographic characteristics were satisfactory predictors of attitudes towards SARs. These results must be cautiously interpreted as our study only included a limited sample of healthcare professionals whereas Bi et al. (40) examined attitudes towards SARs in the Chinese general population, suggesting that cultural difference as well as field of work might partly explain these contradictory results (38, 39, 54).

Acceptance of SARs has been consistently described in the literature as conditional and context-dependent, with favorable attitudes often accompanied by clearly defined constraints related to context of use, perceived relevance, and implementation conditions, rather than reflecting unconditional support (29, 55, 56).

In this sense, favorable attitudes toward SARs do not reflect a generalized endorsement of robotic technologies, but rather a selective evaluation grounded in professional judgment, perceived relevance to care practices, and contextual constraints. Acceptance therefore emerges as a negotiated position that depends on how SARs are expected to function within existing clinical, ethical, and organizational frameworks, rather than as a stable or uniform disposition toward the technology itself.

### 4.6 Perspectives

The results provide several indications for future research on the integration of SARs in healthcare. First, the systematic gap observed between general acceptability and intention to use SARs in one's own practice should be interpreted in light of the cross-sectional survey design, which captures attitudes at a single point in time and relies on hypothetical or self-reported intentions. This format does not allow observation of how attitudes evolve once professionals are exposed to concrete devices, structured training, and organizational constraints. Future longitudinal and implementation studies, for example prospective cohort designs, controlled pilot implementations, or mixed-method implementation studies in specific services, would help determine whether, and under which conditions, initial survey-reported attitudes translate into sustained individual use or, conversely, shift toward more critical stances over time.

Second, the identification of distinct acceptability profiles indicates that attitudes toward SARs are heterogeneous and evolve in relation to specific work contexts rather than following a uniform pattern across professions. Future research could examine how these profiles change over time within particular organizational settings and teams, and how they correspond to concrete implementation stages (e.g., initial introduction, routine use, withdrawal). Mixed-method or qualitative studies following services over time would make it possible to relate changes in acceptability to specific contextual events, such as workload pressures, technical failures, or perceived improvements in care, and to document how professionals justify, adjust, or reconsider the place of SARs in their everyday practice.

Third, the association between prior knowledge of SARs, higher perceived usefulness for psychosocial use cases, and greater emphasis on ethical and organizational safeguards suggests that experience with SARs should be treated as a central analytical dimension rather than a simple covariate. Future work should more precisely characterize different forms of exposure (informational, experimental, and practice-based), including the content, duration, and format of training, and examine how these elements influence professionals' expectations, perceived risks, and implementation requirements over time. In parallel, moving beyond global assessments of “robots in healthcare” toward investigations focused on specific robot models and clearly defined use cases may provide a more fine-grained picture of how acceptability develops for particular practices, and of the concrete challenges that arise when integrating SARs into existing clinical and organizational routines.

From a public-health perspective, elucidating the trajectories of acceptability and adoption of SAR-based practices is essential at a time when technology-mediated care is progressively embedded in health systems. Identifying how contextual factors, professional profiles, and forms of exposure condition the integration of SARs can inform decisions on investment and scaling, the design of regulatory and ethical frameworks, and the development of training and support programs. Such evidence is particularly relevant for anticipating the impact of SAR deployment on workforce organization, safeguarding the rights and well-being of vulnerable populations, and promoting equitable access to technology-supported interventions across services and territories.

### 4.7 Limitations

This study has several limitations related to its methodological framework.

First, the exploratory nature of the study should be acknowledged. The questionnaire was a study-specific instrument designed to explore professionals' perceptions across a broad range of potential SAR use cases and implementation conditions. Although informed by the literature, it was not designed as a standardized psychometric scale. Consequently, the results should be interpreted as exploratory indicators of attitudes rather than definitive measurements.

Second, the study did not collect detailed information about the organizational contexts in which participants worked (e.g., institutional policies, technological infrastructure, or prior exposure to robotics initiatives). Such contextual factors may influence professionals' perceptions and therefore limit the interpretation of the findings.

Third, the professional groups included in the analysis were heterogeneous in size and composition. Some categories were underrepresented, which limits the scope of interprofessional comparisons and suggests that observed differences across professional groups should be interpreted cautiously.

Fourth, the sample was largely composed of professionals working in hospital-based and geriatric settings in a specific national context. This may limit the generalizability of the findings to other healthcare systems, geographical contexts, or specialties where organizational structures, resources, and familiarity with SARs may differ.

Fifth, the cross-sectional survey design captures attitudes at a single point in time and relies on self-reported perceptions and intentions. This design does not allow conclusions about causal relationships or the way acceptability evolves as professionals gain experience with specific SARs, participate in training, or encounter concrete organizational constraints.

Finally, familiarity with SARs was assessed using relatively general self-reported measures of knowledge or exposure. This approach does not allow differentiation by type of robot, duration of exposure, or quality of experience. Consequently, some nuances in the relationship between experience, perceived implementation issues, and acceptability may not have been fully captured.

## 5 Conclusion

In this cross-sectional survey of healthcare professionals in France, the study examined perceived usefulness of SARs across diverse use cases, the importance attributed to implementation-related factors, and the acceptability of integrating SARs into healthcare practice. The data also supported the identification of three distinct attitudinal clusters of respondents, characterized by low, moderate, and high levels of acceptability across use cases and implementation dimensions. Overall, SARs were viewed positively, but acceptability varied according to the type of application and was closely tied to how ethical, organizational, and regulatory conditions were evaluated. Acceptability was more strongly associated with prior knowledge of SARs and attentiveness to ethical and organizational safeguards than with professional category, providing a nuanced understanding of how healthcare professionals position SARs within care practices and under which conditions their integration is considered acceptable, which is essential for planning responsible, context-sensitive deployment in healthcare systems.

## Data Availability

The raw data supporting the conclusions of this article will be made available by the authors, without undue reservation.
