# Supplementary material for: Healthcare professionals’ perspectives on usefulness, acceptability and implementation conditions of socially assistive robots in France: a cross-sectional survey and cluster analysis
Source: Front Digit Health. 2026 Jun 4;8:1802396. doi: 10.3389/fdgth.2026.1802396 (PMC13275396; doi:10.3389/fdgth.2026.1802396)
Supplement: Supplementary file 1 [file Supplementaryfile1.docx]

Supplementary Material


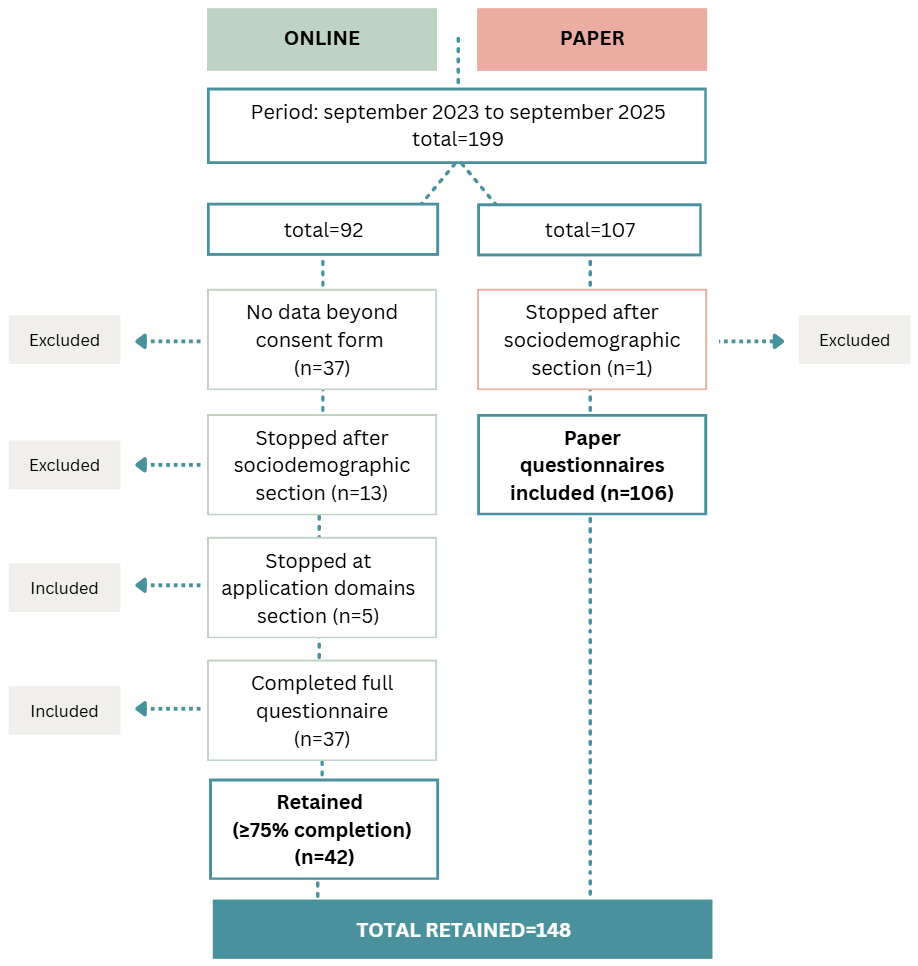


**Supplementary Figure 1.** Flowchart of questionnaire completion and missing data management

| **Variables** | **Correlation (R²)** | **p-value** |
| --- | --- | --- |
| Respecting user autonomy | 0.7387 | 8.616e-27 |
| Preserving user dignity | 0.7284 | 9.577e-26 |
| Ensuring usability | 0.7079 | 8.364e-24 |
| Integration into care practices | 0.6893 | 3.453e-22 |
| Patient and family acceptability | 0.6723 | 8.263e-21 |
| Managing data-related risks | 0.6718 | 9.105e-21 |
| Demonstrating effectiveness | 0.6692 | 1.453e-20 |
| Protecting vulnerable populations | 0.6674 | 1.988e-20 |
| Training healthcare professionals | 0.6569 | 1.230e-19 |
| Psychological support | 0.6540 | 2.020e-19 |
| Cost–benefit balance | 0.6525 | 2.610e-19 |
| Identifying target populations | 0.6428 | 1.281e-18 |
| Professional acceptability | 0.6245 | 2.238e-17 |
| Assisting physical tasks | 0.6157 | 8.189e-17 |
| Implementation costs | 0.6143 | 1.011e-16 |
| Clarifying stakeholder roles | 0.6088 | 2.226e-16 |
| Managing physical risks | 0.6073 | 2.773e-16 |
| Acceptability in healthcare settings | 0.5940 | 1.759e-15 |
| Defining health objectives | 0.5939 | 1.784e-15 |
| Ensuring equitable access | 0.5819 | 8.804e-15 |
| Legal and regulatory frameworks | 0.5805 | 1.045e-14 |
| Recreational activities | 0.5678 | 5.268e-14 |
| Financial viability | 0.5479 | 5.720e-13 |
| Supporting social interaction | 0.5421 | 1.115e-12 |
| Assisting mobility | 0.5059 | 5.411e-11 |
| Supporting learning | 0.4965 | 1.380e-10 |
| Acceptability in own clinical pratical | 0.4567 | 5.391e-09 |
| Psychological support | 0.4441 | 1.563e-08 |
| Assisting communication | 0.4368 | 2.844e-08 |
| Supporting daily routine | 0.4346 | 3.405e-08 |
| Caregiver support | 0.4296 | 5.076e-08 |
| Employment impact | 0.4135 | 1.753e-07 |
| Therapeutic support | 0.4064 | 2.979e-07 |
| Assisting videoconference communication | 0.3449 | 1.769e-05 |
| Helping health monitoring | 0.3048 | 1.659e-04 |
| Assisting feeding | 0.2362 | 3.854e-03 |

**Supplementary Table 1a.** Description of dimension 1 from the principal component analysis

**Supplementary Table 1b.** Description of dimension 2 from the principal component analysis

| **Variables** | **Correlation (R²)** | **p-value** |
| --- | --- | --- |
| Assisting feeding | 0.6706 | 1.137e-20 |
| Caregiver support | 0.6542 | 1.951e-19 |
| Therapeutic support | 0.5484 | 5.415e-13 |
| Assisting mobility | 0.5264 | 6.335e-12 |
| Supporting social interaction | 0.4808 | 6.232e-10 |
| Psychological support | 0.4692 | 1.805e-09 |
| Assisting communication | 0.4600 | 4.071e-09 |
| Supporting learning | 0.4357 | 3.127e-08 |
| Supporting daily routine | 0.4242 | 7.762e-08 |
| Helping health monitoring | 0.4165 | 1.401e-07 |
| Assisting videoconference communication | 0.3353 | 3.103e-05 |
| Assisting physical tasks | 0.3226 | 6.353e-05 |
| Recreational activities | 0.2739 | 7.573e-04 |
| Acceptability healthcare setting | 0.2500 | 2.180e-03 |
| Clarifying stakeholder roles | -0.1657 | 4.409e-02 |
| Financial viability | -0.1683 | 4.086e-02 |
| Implementation costs | -0.1758 | 3.258e-02 |
| Demonstrating effectiveness | -0.1773 | 3.112e-02 |
| Managing data-related risks | -0.1944 | 1.793e-02 |
| Ensuring equitable access | -0.1970 | 1.638e-02 |
| Integration into care practices | -0.2002 | 1.468e-02 |
| Respecting user autonomy | -0.2079 | 1.123e-02 |
| Preserving user dignity | -0.2148 | 8.735e-03 |
| Professional acceptability | -0.2301 | 4.898e-03 |
| Managing physical risks | -0.2365 | 3.802e-03 |
| Protecting vulnerable populations | -0.2451 | 2.674e-03 |
| Employment impact | -0.2560 | 1.686e-03 |
| Patient and family acceptability | -0.2634 | 1.218e-03 |
| Legal and regulatory frameworks | -0.2775 | 6.383e-04 |
| Ensuring usability | -0.3103 | 1.239e-04 |
| Cost–benefit balance | -0.3161 | 9.091e-05 |
| Managing psychological risks | -0.3340 | 3.336e-05 |

**Supplementary Table 2.** Eigenvalue and cumulative percentage of variance

|  | | **Eigenvalue** | | **% of the variance** | | **Cumulative %** | |
| --- | --- | --- | --- | --- | --- | --- | --- |
| Dim. 1 |  | 11.81 |  | 32.80 |  | 32.80 |  |
| Dim. 2 |  | 4.07 |  | 11.30 |  | 44.10 |  |
| Dim. 3 |  | 2.14 |  | 5.94 |  | 50.04 |  |
| Dim. 4 |  | 1.71 |  | 4.74 |  | 54.78 |  |
| Dim. 5 |  | 1.50 |  | 4.18 |  | 58.95 |  |
| Dim. 6 |  | 1.22 |  | 3.40 |  | 62.35 |  |
| Dim. 7 |  | 1.13 |  | 3.13 |  | 65.48 |  |
| Dim. 8 |  | 1.02 |  | 2.82 |  | 68.30 |  |
| Dim. 9 |  | 0.93 |  | 2.59 |  | 70.89 |  |
| Dim. 10 |  | 0.83 |  | 2.31 |  | 73.20 |  |
| Dim. 11 |  | 0.81 |  | 2.25 |  | 75.46 |  |
| Dim. 12 |  | 0.77 |  | 2.14 |  | 77.60 |  |
| Dim. 13 |  | 0.74 |  | 2.07 |  | 79.66 |  |
| Dim. 14 |  | 0.65 |  | 1.81 |  | 81.47 |  |
| Dim. 15 |  | 0.60 |  | 1.66 |  | 83.13 |  |
| Dim. 16 |  | 0.56 |  | 1.55 |  | 84.68 |  |
| Dim. 17 |  | 0.50 |  | 1.40 |  | 86.08 |  |
| Dim. 18 |  | 0.50 |  | 1.39 |  | 87.47 |  |
| Dim. 19 |  | 0.48 |  | 1.32 |  | 88.79 |  |
| Dim. 20 |  | 0.40 |  | 1.12 |  | 89.91 |  |
| Dim. 21 |  | 0.39 |  | 1.08 |  | 90.99 |  |
| Dim. 22 |  | 0.36 |  | 1.00 |  | 91.99 |  |
| Dim. 23 |  | 0.33 |  | 0.91 |  | 92.89 |  |
| Dim. 24 |  | 0.32 |  | 0.90 |  | 93.79 |  |
| Dim. 25 |  | 0.30 |  | 0.82 |  | 94.62 |  |
| Dim. 26 |  | 0.29 |  | 0.80 |  | 95.42 |  |
| Dim. 27 |  | 0.25 |  | 0.69 |  | 96.11 |  |
| Dim. 28 |  | 0.24 |  | 0.67 |  | 96.78 |  |
| Dim. 29 |  | 0.21 |  | 0.58 |  | 97.36 |  |
| Dim. 30 |  | 0.19 |  | 0.53 |  | 97.89 |  |
| Dim. 31 |  | 0.17 |  | 0.47 |  | 98.36 |  |
| Dim. 32 |  | 0.15 |  | 0.42 |  | 98.78 |  |
| Dim. 33 |  | 0.14 |  | 0.38 |  | 99.16 |  |
| Dim. 34 |  | 0.12 |  | 0.33 |  | 99.49 |  |
| Dim. 35 |  | 0.11 |  | 0.30 |  | 99.79 |  |
| Dim. 36 |  | 0.08 |  | 0.21 |  | 100.00 |  |
|  | | | | | | | |

**Supplementary material 1.** Description of AI-Assisted Usages

The generative artificial intelligence tool (Perplexity, GPT‑5.1) was employed exclusively for two types of editorial assistance, as detailed below. In all cases, the tool’s outputs were manually reviewed, verified, and approved by the authors before inclusion in the final manuscript.

1. Editing and stylistic refinement
The tool assisted in improving grammar, language clarity, and adherence to academic style guidelines. It was applied to enhance readability and consistency across sections written by different co-authors, without altering the scientific meaning, results, or interpretations.

Initial prompt:
*“Please review the text below to improve its grammar, clarity, and flow. Maintain all technical terms and the scientific meaning exactly as written. The goal is to adapt the language to a concise and formal academic style suitable for journal submission.”*
Final prompt:
*“Check the revised version of the manuscript section below for any residual grammatical inconsistencies or formatting issues. Do not alter the scientific content or interpretive statements , only adjust for readability and tone consistent with formal academic writing.”*

2. Translation support
The tool was used to translate sections originally drafted in French into English, ensuring consistency of terminology and scientific precision. The translations maintained the original conceptual and technical content, and were subsequently checked and refined by the authors to guarantee accuracy and tone alignment.

Initial prompt:
*“Translate the following text from French to English, ensuring precise preservation of all scientific terms, numerical data, and conceptual meaning. Use formal academic English appropriate for journal publication without altering the content.”*
Final prompt:
*“Review the translated text below to confirm grammatical accuracy and consistency in academic tone. Verify that no scientific meaning, data, or interpretation has been changed during translation.”*
